# Quantum coherence of multiple excitons governs absorption cross-sections of PbS/CdS core/shell nanocrystals

**DOI:** 10.1038/s41467-018-05698-0

**Published:** 2018-08-09

**Authors:** Hirokazu Tahara, Masanori Sakamoto, Toshiharu Teranishi, Yoshihiko Kanemitsu

**Affiliations:** Institute for Chemical Research, Kyoto University, Uji, Kyoto 611-0011 Japan

## Abstract

Multiple excitons in semiconductor nanocrystals have been extensively studied with respect to unique carrier dynamics including quantized Auger recombination and implementation in optoelectronic devices such as solar cells and photodetectors. However, the generation mechanism of multiple excitons still remains unclear. Here, we study instantaneous and delayed multiple exciton generation processes in PbS/CdS core/shell nanocrystals. The absorption cross-sections of biexcitons and triexcitons are identical to that of single excitons under instantaneous excitation with a single pulse. In contrast, the delayed excitation using double pulses shows a reduction of the biexciton and triexciton absorption cross-sections. Our theoretical analysis reveals that the excitonic coherence assists the generation of multiple excitons and that the reduction of multiple exciton absorption cross-sections is caused by the reduction of coherent excitation pathways. We clarify that exciton coherences play a key role in multiple exciton generation processes and seamlessly connect the identical and reduced multiple exciton absorption cross-sections.

## Abstract (web summary)

Understanding the physics underlying the generation and recombination of excitons is expected to help improve the performance of solar call and photodetector devices. Here the authors demonstrate that the quantum coherence of excitons significantly affects multiexciton absorption cross-sections.

## Introduction

Semiconductor nanocrystals (NCs) are being investigated extensively because of their fascinating optoelectronic properties. Their electronic wavefunctions and energy levels are tunable via NC-size control and their hetero-structure design enables highly emissive NCs that can be used in photonic applications such as light-emitting diodes and lasers^1,2^. Furthermore, unique quantum characteristics of NCs such as efficient Auger recombination of excitons and carrier multiplication^3–9^ have been intensively investigated because their understanding is important for use in solar cells and photodetectors^10–12^.

Multiple excitons in NCs have been mainly investigated from the viewpoint of relaxation dynamics. The most important aspect is that the multiple exciton lifetime obeys a scaling law, i.e., it is determined by the number of Auger recombination pathways in the NC^4,13^. In contrast, the details of carrier multiplication, i.e., the multiple exciton generation by a single high-energy photon, and the corresponding absorption dynamics are still under discussion. The multiple exciton absorption cross-sections are usually treated as being independent of the number of excitons and a single value has been used for the cross-sections of excitons, biexcitons and triexcitons in many previous works^14–16^. However, since the absorption coefficient is determined by the strength of the dipole transition between initial and final states, the absorption cross-sections should depend on the number of occupied electrons and holes via strong many-body interactions in NCs. The efficient Auger recombination in NCs is evidence of strong many-body interactions and a deviation from the Poisson model has been discussed for multiple excitons^17^. Furthermore, it has been reported that multiple exciton absorption cross-sections are altered by state filling^18^. Both the model with identical and that with altered absorption cross-sections are used, depending on the experiments. Therefore, it is a long-standing issue to be solved in the NC research field whether absorption cross-sections of multiple excitons depend on the number of excitons or not.

One of the reasons why generation processes of multiple excitons are still not understood sufficiently is that most studies on multiple excitons have been performed under high-energy excitation. Since in this case multiple excitons are generated from high-energy states via energy relaxation, information on the multiple exciton absorption is obscured by relaxation processes. Thus, the direct generation of multiple excitons via multiphoton absorption for the lowest energy state, i.e., excitation at exciton resonance, is required to determine the different cross-sections. Under resonant excitation, multiple excitons can be generated coherently by excitonic dipole transitions. Besides several unique behaviors of coherent excitons in NCs^19,20^, the multiple exciton coherences are also considered to be important in generation processes of multiple excitons^21–23^. However, the analysis of coherent dynamics such as harmonic quantum coherence of multiple excitons^24^ cannot be performed with the standard method that monitors only the number of photogenerated carriers. Therefore, comprehensive analysis including both aspects, the number and the coherence of excitons, is required to understand the fundamental generation processes of multiple excitons.

In this study, we report the effect of multiple exciton coherence on the absorption cross-section in semiconductor NCs. We performed transient absorption (TA) measurements in single and double pump-pulse configurations to determine instantaneous and delayed generation processes of multiple excitons via multiphoton absorption, respectively. Comparing the results obtained for both generation processes, we investigated the effect of the harmonic quantum coherence of multiple excitons, i.e., the harmonic dipole oscillations, which occurs predominantly in the instantaneous generation process. While identical absorption cross-sections were obtained for single pulse excitation, the absorption cross-sections obtained for double pulse excitation varies. The impact of the excitonic coherence can be clarified with the difference between both experiments. The increase in the number of excitons per NC was monitored with the excitation-fluence dependence of the TA dynamics. The analysis of these data allowed us to establish a general law for the generation efficiency of multiple excitons. This work evidences that multiple exciton coherence is the key for efficient generation of multiple excitons in NCs.

## Results

### Multiple exciton dynamics

**Fig. 1:**
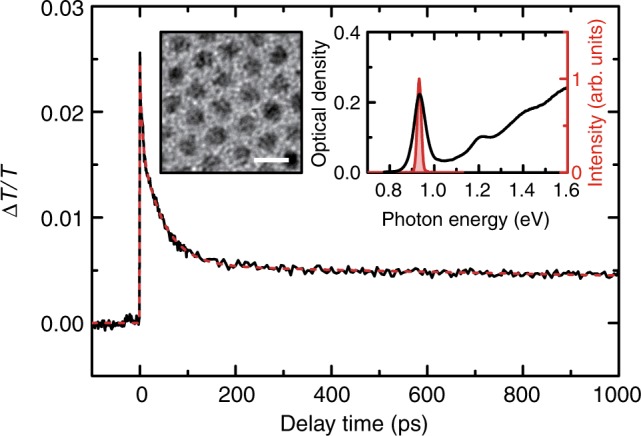
Multiple exciton dynamics in a single pump-pulse measurement. Experimental (black solid curve) and calculated (red dotted curve) results of TA dynamics in PbS/CdS core/shell NCs for excitation with a single pump pulse. The insets show the transmission electron microscope image, scale bar: 10 nm, and the absorption spectrum of the nanocrystal sample. The spectrum of the pump pulse is shown with the red shaded area

The sample used in this study was colloidal PbS/CdS core/shell NCs dispersed in toluene, which were synthesized according to a method described in previous investigations^24–26^. The transmission electron microscope image and absorption spectrum of the NCs are shown in the insets of Fig. 1. The average diameter of the core/shell NCs is 5.2 ± 0.1 nm. Note that the diameter of the PbS core is slightly smaller. The NCs exhibit a strong excitonic absorption peak at 0.93 eV. To characterize the decay dynamics of the multiple excitons in the NCs, we performed standard TA measurements with single pump-pulse excitation, where the photon energy of pump and probe pulses was tuned to the excitonic absorption peak. The result of the TA measurement is shown with the black solid curve in Fig. 1. We observed a fast decay within the first 200 ps followed by a signal with long lifetime. The almost constant signal is assigned to the photobleaching induced by single excitons, since the lifetime of single excitons in PbS NCs has been reported to be a few hundred nanoseconds^14^, which is much longer than the measurement range of our experiments. In contrast, the fast decay is a result of Auger recombination of multiple excitons^3–7,14^. To obtain the lifetimes of the multiple excitons, the TA signal was fitted by a triple exponential decay function: $\Delta T/T(t) = \mathop {\sum }\nolimits_{i \hskip 2pt = \hskip 2pt 1,2,3} A_i{\mathrm e}^{ - t/\tau _i}$, where *A*_*i*_ and *τ*_*i*_ are the amplitude and lifetime of the *i*th state (*i* = 1,2,3 correspond to single exciton, biexciton, and triexciton), respectively. This is the standard analysis for multiple exciton decays^3,14,16^. As shown with the red dotted curve in Fig. 1, the calculated result is in good agreement with the experimental result. From the fitting we obtained 47 and 3.9 ps for the lifetimes of biexcitons and triexcitons, respectively. These lifetimes are used as constants for the discussion of our results below.

### TA measurements by single and double pump-pulse excitation

**Fig. 2:**
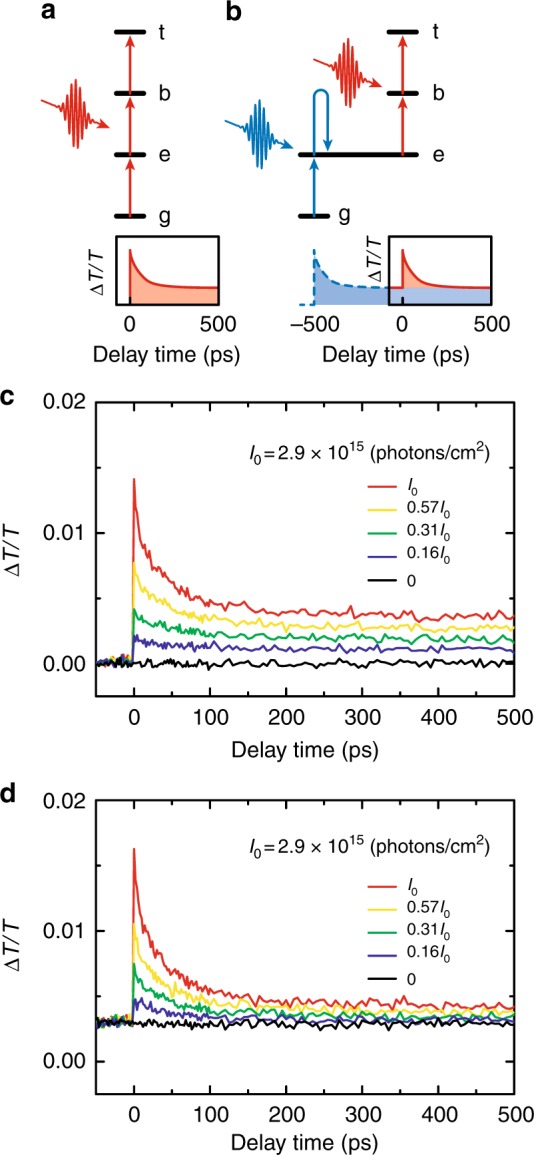
Excitation diagrams and TA dynamics in single and double pump-pulse measurements. **a**, **b** Excitation diagrams for **a** single and **b** double pump-pulse experiments. The unoccupied ground state, single exciton, biexciton, and triexciton states are denoted by g, e, b, and t, respectively. **c**, **d** Pump-fluence dependence of the TA dynamics under **c** single and **d** double pump-pulse excitation. In the double pulse excitation, the first pump pulse had a constant fluence of 0.52*I*_0_ and excited the sample at the delay time of −500 ps. The photon fluences of the pump pulse that excites the sample at 0 ps are shown in the figures

To understand the generation mechanisms of multiple excitons, we compared the results of TA measurements using single and double pump-pulse excitation. The excitation diagrams for the single and double pump-pulse excitation are shown in Fig. 2a, b, respectively. In the single pulse excitation, multiple excitons including biexcitons and triexcitons are generated instantaneously from the unoccupied ground state. Since the transition from the unoccupied ground state to the single exciton and that from the single exciton to the multiple exciton occur coherently within the laser pulse duration, the single pump-pulse measurement reflects the coherent state of the single exciton as an intermediate state. This coherent excitation condition is preserved during the pulse duration, even if the excitonic coherence time is shorter than the pulse duration, since the excitonic dipole oscillation is continuously driven by the electric field of the excitation pulse. In contrast, the excitation with double pump pulses (shown in Fig. 2b) generates multiple excitons via temporally separated transitions: the first step is the transition from the unoccupied ground state to a single exciton state, which also includes energy relaxation from a multiple exciton state to a single exciton state, and the second step is the transition from the single exciton state to a multiple exciton state. The time interval between the first and second excitation pulses was adjusted to 500 ps in this experiment. The excitonic coherence generated by the first pump pulse cannot be preserved until the arrival of the second pump pulse, since the excitonic coherence time at room temperature is comparable to or shorter than the pulse duration^27^. The multiple excitons generated by the first pump pulse undergo recombination processes leading to a single exciton state before the second pulse arrives, because the Auger recombination lifetime of biexcitons is a few tens of picoseconds. Consequently, most of multiple excitons are generated from an incoherent single exciton in the delayed excitation process. The comparison between the single and double pump-pulse TA dynamics clarifies the influence of the excitonic coherence on the generation processes of multiple excitons via multiphoton absorption.

The lower panels of Fig. 2a, b show the schematics of single and double pump-pulse TA measurements, respectively. In contrast to the standard TA measurement with a single pump pulse, the double pump-pulse TA measurement can control the initial state for the multiple exciton generation that occurs with the second pulse. In the double pulse excitation, the time origin was defined with the arrival time of the second pump pulse. To prepare an incoherent single exciton at the time origin, we tuned the time interval between the two pump pulses to 500 ps as shown in the lower panel of Fig. 2b. We note that similar TA signals are also observed for longer time intervals, since the exciton lifetime is much longer than 500 ps. This time separation is sufficiently long to allow complete relaxation of the multiple excitons, because the lifetimes of biexcitons and triexcitons are much shorter than 500 ps.

The dependences of the TA dynamics on the photon fluence of the pump pulse at the time origin under single and double pump-pulse excitation are shown in Fig. 2c, d, respectively. With increasing excitation-photon fluence, biexcitons and triexcitons are clearly observed in both experiments. By using the same fitting procedure as explained for Fig. 1, we verified that the lifetimes of biexcitons and triexcitons are independent of the excitation-photon fluence, while the amplitudes of single excitons, biexcitons, and triexcitons increase with the fluence. The subtle differences between the two measurements are important for the understanding of the generation mechanisms of multiple excitons. The TA signals at negative delay times are caused by the single exciton state that was prepared by the first pump pulse.

These single and double pump-pulse experiments show similar multiple exciton decay dynamics, but they reflect the difference of the excitation sequence. Under single pump-pulse excitation, multiple excitons are directly generated from the unoccupied ground state. Thus, *n* excitons in one NC are generated by absorption of *n* photons. In contrast, under double pump-pulse excitation, *n* excitons in one NC are generated from a single exciton state by absorption of *n* − 1 photons if a single exciton state has been generated by the first pump pulse as it can be achieved by setting the time interval between the two pump pulses to 500 ps. Then we can understand the state-selective generation processes of multiple excitons using the difference in the numbers of excitons and photons.

**Fig. 3:**
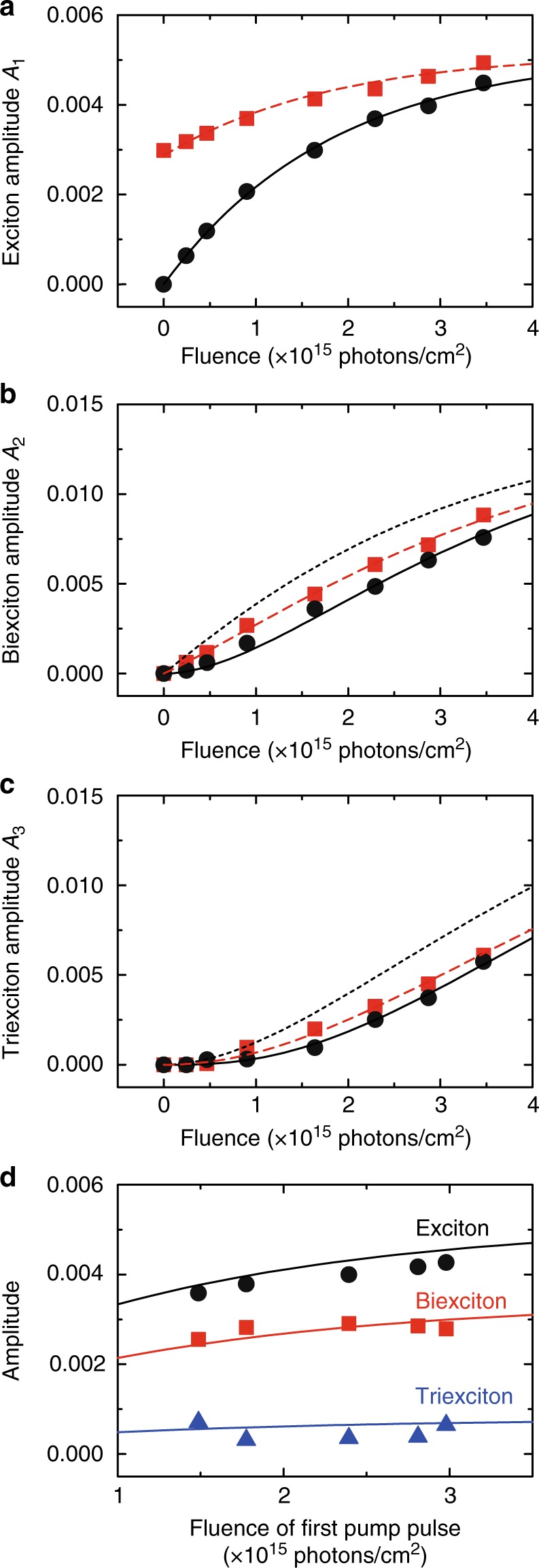
TA amplitudes for single and double pump-pulse excitation. **a**–**c** TA amplitudes of **a** single excitons, **b** biexcitons, and **c** triexcitons. The fitting results of these amplitudes for single and double pump-pulse excitation are plotted by the black filled circles and red filled squares, respectively. The calculated results for the single pump-pulse experiments are shown by the black solid curves. The calculated results for the double pump-pulse experiments under the assumptions of identical and variable absorption cross-sections are shown by the black dotted and red dashed curves, respectively. **d** Dependence of TA amplitudes on the first pump-pulse fluence for double pump-pulse excitation. The solid curves are calculated using the absorption cross-sections obtained from Fig. 3a–c

The difference between single and double pump-pulse excitation processes can be clarified by analyzing the multiple exciton absorption cross-sections, since they directly reflect the generation efficiencies of multiple excitons. The absorption cross-sections of single excitons, biexcitons, and triexcitons can be determined by the excitation-fluence dependence of the TA amplitudes. The amplitudes of single excitons, biexcitons, and triexcitons obtained under single pump-pulse excitation are plotted with the black filled circles in Fig. 3a–c, respectively. Figure 3a clearly shows a saturation behavior in the amplitude of single excitons. This saturation behavior under high excitation-photon fluence shows that almost all NCs are excited. The exciton amplitudes obtained for double pump-pulse excitation (red filled squares in Fig. 3a) also show an increase with photon fluence and saturate as well. The data of the biexcitons and triexcitons in Fig. 3b, c exhibit no saturation at the highest fluences used in this experiment. Overall, the TA amplitudes in the double pump-pulse experiments are larger than those in the single pump-pulse experiments, since a single exciton has been already prepared by the first pump pulse.

### TA amplitudes for single pump-pulse excitation

The dependence of the TA amplitudes on the photon fluence of the pump pulse at the time origin can be analyzed by considering the distribution of the number of absorbed photons, which is described by the Poisson distribution. Below we discuss the TA amplitudes obtained under single pump-pulse excitation on the basis of the standard analysis of absorption cross-sections^4,14–16^. The Poisson distribution, $P_n(\langle N\rangle ) = \langle N\rangle ^n{\mathrm e}^{ - \langle N\rangle }/n!$, describes the absorption probability of a certain number of photons, *n*, for an average number of absorbed photons, $\langle N\rangle$. Since absorption of at least *n* photons is required to generate *n* excitons, the generation probability of *n* excitons is expressed by ${\mathrm{\Sigma }}_{i \ge n}P_i(\langle N\rangle )$. Using this generation probability, the fluence dependences of the amplitudes for single excitons (*A*_1_), biexcitons (*A*_2_), and triexcitons (*A*_3_) can be written

1 $$A_1(J) = A_0^{({\mathrm{X}})}\{ 1 - P_0(\sigma J)\} ,$$

2 $$A_2(J) = A_0^{\left( {2{\mathrm{X}}} \right)}\{ 1 - P_0(\sigma J) - P_1(\sigma J)\} ,$$

3 $$A_3(J) = A_0^{\left( {3{\mathrm{X}}} \right)}\{ 1 - P_0(\sigma J) - P_1(\sigma J) - P_2(\sigma J)\} ,$$

where *σ* and *J* are the absorption cross-section and photon fluence, respectively. Here, we use a single value for the absorption cross-sections of excitons, biexcitons and triexcitons, which is justified later. The average number of excitons is determined by the product of the absorption cross-section and the photon fluence, i.e., $\langle N\rangle$ = *σJ*. The distribution *P*_0_(*σJ)* describes therefore the fraction of unexcited NCs for the pump pulse with the photon fluence *J*. The saturated amplitudes are denoted by $A_0^{({\mathrm{X}})}$, $A_0^{\left( {2{\mathrm{X}}} \right)}$, and $A_0^{\left( {3{\mathrm{X}}} \right)}$ for the single exciton, biexciton, and triexciton components, respectively. The fitting results are *σ* = (5.4 ± 0.3) × 10^−16^ cm^2^, $A_0^{({\mathrm{X}})} = (5.2 \pm 0.2) \times 10^{ - 3}$, $A_0^{\left( {2{\mathrm{X}}} \right)} = (13.9 \pm 0.3) \times 10^{ - 3}$, and $A_0^{\left( {3{\mathrm{X}}} \right)} = (19.1 \pm 0.4) \times 10^{ - 3}$ and the calculated curves are shown with the black solid curves in Fig. 3. The good agreement with the experimental results implies that the absorption cross-sections of biexcitons and triexcitons are in fact identical to that of single excitons in the single pump-pulse experiments.

We note that the absorption cross-section obtained from the above analysis agrees well with that determined from a previous study of PbS NCs^28^, which investigated the exciton peak energy and absorption cross-section as a function of the NC size. Using their NC size dependence and the exciton peak energy of our sample, the absorption cross-section is calculated to be 5.6 × 10^−16^ cm^2^. This is consistent with our abovementioned result and confirms the high reliability of the values obtained from the TA measurements. Furthermore, the generation probabilities of biexcitons and triexcitons are also reproduced by the same absorption cross-section. We consider that the high degeneracy of the lowest exciton states in PbS NCs, i.e., 8-fold degeneracy^29^, allows us to perform the analysis using the Poisson distribution even under high excitation-photon fluence. This would not be possible in the case of 2-fold degenerate systems such as CdSe NCs^30^.

### TA amplitudes for double pump-pulse excitation

Next, we discuss the TA amplitudes that were obtained from the double pump-pulse experiments. Here, the contribution of the first pump pulse, which excited the NCs 500 ps before the second pump, has to be considered. The first pump pulse excites most of the NCs and then Auger recombination of multiple excitons occurs, resulting in NCs occupied with single excitons. However, a part of the NCs remains unexcited. The ratio between the excited and unexcited NCs after the first pump is determined by the Poisson distribution of the number of absorbed photons, $1 - P_0\left( {\langle N_0\rangle } \right):P_0\left( {\langle N_0\rangle } \right)$, where the average number of excitons generated by the first pump pulse is denoted by $\langle N_0\rangle$. Thus, the TA amplitude of the single excitons in the double pump-pulse TA measurement is expressed by

4 $$A_1(J) = \left\{ {1 - P_0\left( {\langle N_0\rangle } \right)} \right\}A_0^{\left( {\mathrm{X}} \right)} + P_0\left( {\langle N_0\rangle } \right)A_0^{\left( {\mathrm{X}} \right)}\{ 1 - P_0(\sigma J)\} .$$

The first term in Eq. (4) determines the number of single excitons generated by the first pump pulse, while the second term expresses the additional single excitons generated by the second pump pulse. The saturated amplitude $A_0^{\left( {\mathrm{X}} \right)}$ is the same value as that determined in the single pump-pulse experiment. From the fitting with Eq. (4), we obtain the initial number of excitons $\langle N_0\rangle = 0.81 \pm 0.02$, which means that the first pump pulse generates 0.81 excitons per NC on average, and the calculated result is shown by the red dashed curve in Fig. 3a. The agreement with the experimental data is excellent, which confirms the validity of our model. In analogy to Eq. (4), the TA amplitudes of biexcitons and triexcitons in the double pump-pulse experiment can be expressed by

5 $$\begin{array}{l}A_2(J) = \left\{ {1 - P_0\left( {\langle N_0\rangle } \right)} \right\}A_0^{\left( {2{\mathrm{X}}} \right)}\{ 1 - P_0(\sigma{\prime} J)\}\\ + P_0\left( {\langle N_0\rangle } \right) A_0^{\left( {2{\mathrm{X}}} \right)}\{ 1 - P_0(\sigma J) - P_1(\sigma J)\}, \end{array}$$

6 $$\begin{array}{l}A_3\left( J \right) = \left\{ {1 - P_0\left( {N_0} \right)} \right\}A_0^{\left( {3{\mathrm X}} \right)}\left\{ {1 - P_0\left( {\sigma{\prime\prime} J} \right) - P_1\left( {\sigma{\prime\prime} J} \right)} \right\}\\ + P_0\left( {\langle N_0\rangle } \right)A_0^{\left( {3{\mathrm X}} \right)}\{ 1 - P_0(\sigma J) - P_1(\sigma J) - P_2(\sigma J)\} ,\end{array}$$

where the saturated amplitudes of biexciton ($A_0^{\left( {2{\mathrm{X}}} \right)}$) and triexciton ($A_0^{\left( {3{\mathrm{X}}} \right)}$) are those determined in the single pump-pulse measurement. In these equations, the first and second terms describe the TA signals caused by the photoabsorption processes from NCs that contain a single exciton and unoccupied NCs, respectively. The absorption cross-sections of the transitions from the single exciton state to the biexciton and triexciton states are denoted by *σ*′ and *σ*′′, respectively. These cross-sections govern the transitions that start from the incoherent single exciton state. The most important issue of this work is to clarify whether the multiple exciton absorption cross-sections *σ*′ and *σ*′′ are equal to *σ* or not. The black dotted curves in Fig. 3b, c show the predicted results of the TA amplitudes by assuming identical cross-sections, i.e., *σ* = *σ*′= *σ*′′. In this calculation there is no variable parameter, because the parameters were already determined in the above analyses. This assumption of identical cross-sections is plausible, since any changes in multiple exciton absorption cross-sections are not observed when the double pump-pulses overlap partially or fully^24^. In contrast, the experimental results of biexciton and triexciton amplitudes (red filled squares in Fig. 3b, c) are significantly smaller than the calculated black dotted curves. Therefore, the assumption of identical multiple exciton cross-sections is not valid in the case of transitions from incoherent single excitons. The difference between the experimental and calculated results indicates that the generation probabilities of biexcitons and triexcitons are influenced by excitonic coherence, since the excitation diagrams of these experiments are almost the same except for the difference in the coherence of the intermediate single exciton state.

To describe the measured TA amplitudes of the multiple excitons, we fitted them by using Eq. (5) and (6) under the assumption of different absorption cross-sections. The fitting results are shown with the red dashed curves in Fig. 3c, d. The good agreement between the predicted trend and the experimental data indicates that our fitting model with different multiple exciton absorption cross-sections is correct in the case of transitions from incoherent single exciton states. Since the calculations are performed for a single variable parameter *σ*′ or $\sigma{\prime\prime}$, the absorption cross-sections can be obtained with high accuracy. The estimated absorption cross-section in the transition from the incoherent single excitons to biexcitons is *σ*′ = (3.2 ± 0.1) × 10^−16^ cm^2^, which is significantly smaller than *σ* = (5.4 ± 0.3) × 10^−16^ cm^2^. The estimated triexciton absorption cross-section, $\sigma{\prime\prime} = (3.6 \pm 0.1) \times 10^{ - 16}$ cm^2^, is also smaller than *σ*. The absorption cross-section of the biexciton, *σ*′, is slightly smaller than that of the triexciton, $\sigma{\prime\prime}$, but the difference is too small to decide whether they are actually equal or not. We note that the fitting results are similar, even if we assume $\sigma{\prime} = \sigma{\prime\prime}$. Thus, theoretical analysis is required to discuss the details. A simple model explained later can describe the slight difference.

Our result that the reduction of the absorption cross-sections is determined by intrinsic processes is further supported by the dependence of the TA amplitudes on the first pump-pulse fluence. If the change in the absorption cross-sections is originated from extrinsic factors such as trap states, the increase of photons absorbed during the first pump pulse drastically changes the trap conditions; the TA signals should deviate from the model represented by Eq. (4)–(6). On the other hand, if the dependence on the first pump-pulse fluence follows the curve calculated by our model, we can confirm that extrinsic factors do not contribute to the reduction of the absorption cross-sections. Figure 3d shows the dependence of the TA amplitudes on the first pump-pulse fluence for double pump-pulse excitation. Here, the fluence of the second pump pulse was set to 0.90 × 10^15^ photons/cm^2^. The solid curves show the calculated results drawn by Eq. (4)–(6), where we use the saturated amplitudes and absorption cross-sections determined from Fig. 3a–c. The theoretically predicted curves reproduce the trends of the experimental results fairly well. This suggests that extrinsic factors are not dominant in the experiments and that our model accurately describes the double pump-pulse experiments.

### Absorption cross-sections of multiple excitons

**Fig. 4:**
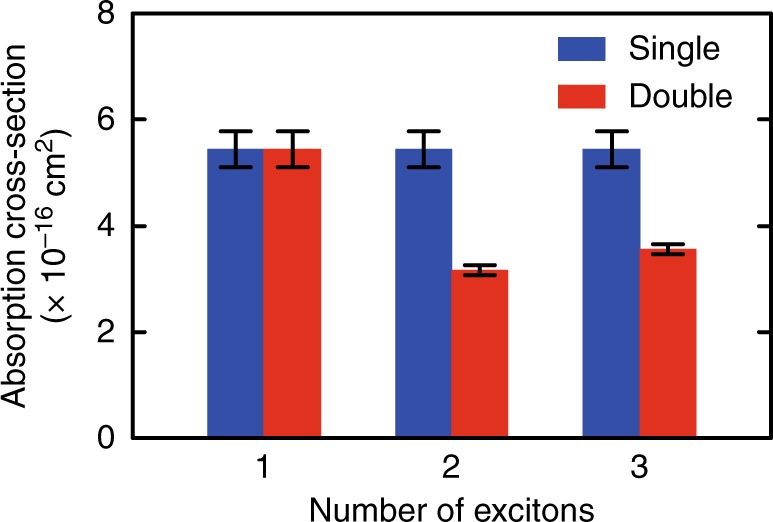
Absorption cross-sections under single and double pump-pulse excitation conditions. The absorption cross-sections are shown for the single excitons, biexcitons, and triexcitons. Error bars represent the uncertainty in the determination of the absorption cross-sections

The absorption cross-sections obtained in this work are summarized in Fig. 4. Under the single pump-pulse condition (blue bars in Fig. 4), the absorption cross-sections were independent of the number of excitons, which has also been frequently observed in previous investigations^14–16^. In contrast, the multiple exciton absorption cross-sections strongly decrease under the double pump-pulse condition (red bars in Fig. 4). The decrease of the multiple exciton absorption cross-sections is beyond the framework of the simple assumption of identical absorption cross-sections for multiple excitons. The decrease indicates that the excitonic coherence plays an important role in the excitation processes. As shown in Fig. 2a, b, the excitation diagrams of the single and double pump-pulse experiments are almost the same except for the absence of the excitonic coherence in the latter. Since the excitonic coherence disappears within the ultrafast time regime, it was not possible to observe the time evolution of the multiple exciton absorption cross-sections. However, our result clearly shows the difference in the generation processes of multiple exciton states via coherent and incoherent excitons. We note that the decrease of the multiple exciton absorption cross-sections in the double pump-pulse excitation is not a result of ultrafast carrier dynamics in the first excitation process. Carrier upconversion due to Auger recombination and bandgap shift due to hot-carrier cooling can occur immediately after the first excitation^6,31^, but these ultrafast carrier dynamics are not relevant at the time when the strongly delayed second pump pulse arrives. Therefore, the difference between the absorption cross-sections of single and double pump-pulse experiments indicates that the efficient generation processes of multiple excitons are assisted by excitonic coherence.

## Discussion

**Fig. 5:**
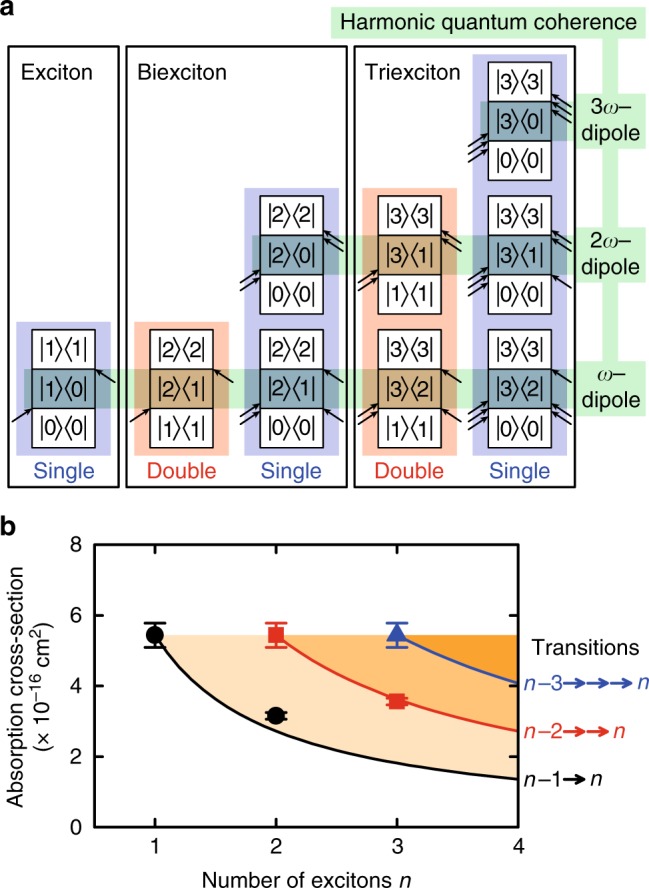
Excitation sequences and correlation of the absorption cross-section with exciton number. **a** Double-sided Feynman diagrams of single exciton, biexciton, and triexciton generation processes. **b** Correlation between absorption cross-section and number of excitons. The theoretically predicted absorption cross-sections are shown with the solid curves, which depend on the transitions indicated on the right side of the figure. Error bars represent the uncertainty in the determination of the absorption cross-sections

The efficient multiple exciton generation process during the single pump-pulse excitation can be understood by discussing multiple exciton coherences using double-sided Feynman diagrams^24,32–34^. The excitation sequences for single and double pump-pulse excitation are expressed by the evolution of the double-sided diagram $|i\rangle \langle j|$ (Fig. 5a), which corresponds to the density matrix *ρ*_*ij*_ in optical Bloch equations^32,33^. Here, the state composed of *n* excitons is denoted by $|n\rangle$. Since TA measurements allow us to determine the number of excitons, we discuss excitation pathways that reach diagonal elements $|n\rangle \langle n|$, i.e., population terms in the density matrix. The off-diagonal element $|n\rangle \langle m|$ (*m*≠*n*) denotes the dipole between the $|n\rangle$ and $|m\rangle$ states, which assists the photoexcitation from the $|m\rangle$ state to the $|n\rangle$ state. Figure 5a shows the different possible excitation pathways to reach the single exciton, biexciton, and triexciton populations. Here, we note that the pathways become more complicated if the multiple exciton’s fine structure is taken into account. In our analysis, we assume that the difference in the fine-structure states is negligibly small at room temperature so that we can discuss the effective pathways. The initial state in the single pump-pulse excitation is the unoccupied ground state $|0\rangle \langle 0|$, while the initial state for the second pump in the double pump-pulse excitation is the single exciton state $|1\rangle \langle 1|$ that was prepared by the first pump pulse. The different initial state causes a different number of available excitation pathways as shown in Fig. 5a. The frequency-multiplied dipoles $|2\rangle \langle 0|$ and $|3\rangle \langle 0|$ are available in the transitions from $|0\rangle \langle 0|$. In contrast, since these dipoles are not generated from the single exciton state $|1\rangle \langle 1|$, the possible excitation pathways in the sequential excitation are reduced. The reduction of the number of excitation pathways is directly related to the decrease of the measured absorption cross-section.

Quantitative discussion is essential for practical application of our results, and therefore we calculated the absorption cross-sections on the basis of the double-sided Feynman diagrams. In the single pump-pulse experiment, the absorption cross-section including all excitation pathways via harmonic quantum coherences is independent of the number of excitons. Here, we define *σ*_0_ as the absorption cross-section that includes all excitation pathways indicated by the blue shaded area for each exciton state in Fig. 5a. Since the generation probability of the *n*th-order harmonic quantum coherence, i.e., *nω*-dipole oscillation, is determined by the absorption of *n* or more photons^24^, the increase of the harmonic quantum coherence in the multiphoton absorption can be also explained by Eq. (1)–(3). From the Feynman diagrams, it can be seen that the change of the excitation condition from single to double pulses causes a reduction of the number of excitation pathways from two to one (from three to two) for biexciton (triexciton) generation. Thus, the absorption cross-sections of biexcitons and triexcitons are expected to be *σ*_0_/2 and 2*σ*_0_/3, respectively. For arbitrary transitions, the absorption cross-section of the transition from $|n - m\rangle$ to $|n\rangle$ by *m*-photon absorption can be generally expressed as *mσ*_0_/*n*, where *σ*_0_ is the absorption cross-section of the single exciton state. The theoretically predicted absorption cross-sections for the transitions from $|n - 1\rangle$ to $|n\rangle$, from $|n - 2\rangle$ to $|n\rangle$, and from $|n - 3\rangle$ to $|n\rangle$ are shown with the black, red, and blue solid curves in Fig. 5b, respectively. The good agreement between the experimental and calculated results strongly suggests that the frequency-multiplied dipoles, i.e., harmonic quantum coherences of multiple excitons^24^, assist the generation processes of multiple excitons. The higher-order transitions such as those of the quadexcitons were not observed in our experiment, but their observation can lead to a deeper understanding of the quantized absorption cross-sections predicted in Fig. 5b.

In conclusion, we studied multiple exciton absorption cross-sections by single and double pump-pulse TA measurements. We found that the multiple exciton absorption cross-sections of the transitions starting from coherent excitons are about 1.6 times larger than those of the transitions starting from incoherent excitons. This is the experimental proof that multiple exciton absorption cross-sections can be controlled and improved by excitonic coherences. We proposed a theoretical model that accounts for the harmonic quantum coherences of multiple excitons, which quantitatively predicts the multiple exciton absorption cross-sections depending on the number of excitons. The general law for the multiple exciton absorption cross-sections presented in this work provides deep understanding of the fundamental nature of multiple excitons and can lead to advanced photonic applications including highly efficient photodetectors and solar cells.

## Methods

### Transient absorption measurements

The pump and probe pulses were generated by splitting the output beam from an optical parametric amplifier (OPA) that was pumped via a Ti:sapphire regenerative amplifier with a pulse duration of 100 fs and a repetition rate of 1 kHz. The photon energy of the OPA output beam was tuned to the excitonic absorption peak, and the time delay between the pump and probe pulses was adjusted with a stepper-motor-driven delay stage. We performed TA measurements using single and double pump-pulse excitation. The double pump pulses were generated with an additional beam splitter and the time delay between them was adjusted with a further delay stage. All experiments were performed at room temperature and the NCs were continuously stirred in toluene to remove the influence of photoinduced charging^35^.

### Data availability

The data that support the findings of this study are available from the corresponding authors upon reasonable request.
