# Publisher Correction: Quantum coherence of multiple excitons governs absorption cross-sections of PbS/CdS core/shell nanocrystals

**DOI:** 10.1038/s41467-018-06285-z

**Published:** 2018-09-12

**Authors:** Hirokazu Tahara, Masanori Sakamoto, Toshiharu Teranishi, Yoshihiko Kanemitsu

**Affiliations:** Institute for Chemical Research, Kyoto University, Uji, Kyoto 611-0011 Japan

Correction to: *Nature Communications*; 10.1038/s41467-018-05698-0; published online: 09 Aug 2018

The original version of this Article contained an error in the seventh sentence of the second paragraph of the ‘TA amplitudes for double pump-pulse excitation” section of the Results, which incorrectly read ‘The absorption cross-sections of the transitions from the single exciton state to the biexciton and triexciton states are denoted by σ′ and σ*''*, respectively.’ The correct version states ‘*σ*′′’ in place of ‘σ*''*’.

The ninth and tenth sentences of the same paragraph originally incorrectly read ‘The most important issue of this work is to clarify whether the multiple exciton absorption cross-sections σ′ and σ*''* are equal to σ or not. The black dotted curves in Fig. 3b, c show the predicted results of the TA amplitudes by assuming identical cross-sections, i.e., σ = σ ′ = σ*''* = .’ The correct version states ‘σ′ and *σ*′′ are equal” instead of ‘σ′ and σ*''* are equal’ and ‘σ = σ′ = *σ*′′’ in place of ‘σ = σ ′ = σ*''* = ’.

This has been corrected in both the PDF and HTML versions of the Article.

